# Chronic cerebrospinal venous insufficiency is not associated with cognitive impairment in multiple sclerosis

**DOI:** 10.1186/1741-7015-11-167

**Published:** 2013-07-18

**Authors:** Ralph HB Benedict, Bianca Weinstock-Guttmam, Karen Marr, Vesela Valnarov, Cheryl Kennedy, Ellen Carl, Christina Brooks, David Hojnacki, Robert Zivadinov

**Affiliations:** 1 Department of Neurology, State University of New York at Buffalo, 100 High St, Buffalo, NY 14203, USA; 2 Buffalo Neuroimaging Analysis Center, Department of Neurology, State University of New York at Buffalo, 100 High St, Buffalo, NY 14203, USA; 3 Department of Neurology, School of Medicine and Biomedical Sciences, 100 High St, Buffalo, NY 14203, USA

**Keywords:** Multiple sclerosis, CCSVI, Cognition

## Abstract

**Background:**

Chronic cerebrospinal venous insufficiency (CCSVI) has been reported in multiple sclerosis (MS) yet its significance in relation to cognitive function is undetermined.

This study measured the association between the presence and severity of CCSVI and cognitive impairment in patients with MS.

**Methods:**

CCSVI was assessed using extra-cranial and trans-cranial Doppler sonography in 109 MS patients (79 with relapsing-remitting, 23 with secondary-progressive and 7 with primary-progressive disease subtype). A subject was considered CCSVI-positive if ≥2 venous hemodynamic criteria were fulfilled. The Minimal Assessment of Cognitive Function in MS (MACFIMS) battery was administered assessing the full spectrum of cognitive domains known to be affected by MS. Depression was quantified using the Beck Depression Inventory Fast Screen (BDIFS). Partial correlations, analysis of variance (or covariance) and linear regression were used to examine the hypothesis that CCSVI status is related to cognition or depression after controlling for education and gender.

**Results:**

There were 64 (58.7%) patients who were considered CCSVI-positive. The regression models predicting venous hemodynamic insufficiency severity score were not statistically significant for any of the MACFIMS predictor variables. The analysis of variance tests showed a significant effect of CCSVI-positive diagnosis on cognitive ability in only one of the 10 MACFIMS outcomes, and that one was in the opposite direction of the tested hypothesis. There was no correspondence between CCSVI diagnosis and depression, as measured by the BDIFS.

**Conclusions:**

We find no evidence of an association between the presence and severity of CCSVI with cognitive impairment and depression in patients with MS.

## Background

Multiple sclerosis (MS) is an inflammatory disease of the central nervous system, causing both demyelination and neurodegeneration [1,2]. As would be expected, a substantial number, roughly 50% [3-5], of MS patients have cognitive impairment. In recently diagnosed or benign course patients, the incidence ranges from 20% to 40% [5,6] whereas in samples with a substantial secondary progressive course, roughly 60% of patients are affected [4]. The correlation between cognitive impairment and brain atrophy is robust [7-9]. However, why some patients show cognitive impairment and brain atrophy while others do not is poorly understood.

Chronic cerebrospinal venous insufficiency (CCSVI) was first reported in MS patients in 2009 [10]. As a vascular condition, CCSVI is characterized by anomalies of the main extra-cranial cerebrospinal venous routes, mainly in internal jugular and azygos veins that are hypothesized to interfere with normal venous outflow from the brain to the periphery. Since then, the topic has met with unprecedented controversy following a wide range of reported CCSVI frequencies in MS studies [11-13]. Diagnosis of CCSVI implies a pathological condition the determination of which is based mainly on color Doppler sonography (DS) of extra- (neck) and intra-cranial veins using five venous hemodynamic (VH) criteria (with cutoff of ≥2 positive criteria used for a diagnosis of CCSVI) [10,14]. So far, published studies comparing the prevalence of CCSVI in MS patients and controls [12,15] have not reproduced the original findings of Zamboni *et al*. showing 100% sensitivity/specificity [10,14]. While some groups did report a higher prevalence in MS patients than controls [16,17], others reported the opposite, that is, no greater frequency in MS than in healthy persons [16,18-22]. In the largest cohort studied to date, we found a CCSVI frequency of 56.1% in MS patients compared to 22.7% in healthy controls [23]; however, the condition was also detected at a high frequency in patients with other neurologic diseases.

While not causative, some studies suggest that CCSVI may be a risk factor for clinical worsening in MS [24-26], although here, too, there are contradictory results [16,20]. In a large cohort study exploring the association between CCSVI status and both lesion burden and brain atrophy in MS, no relationship was found [27].

If CCSVI is a risk factor for neurodegeneration or progressive neurologic disability, we would expect significant correlation between CCSVI and cognitive impairment within MS cohorts. The present study was intended to examine this hypothesis.

## Methods

### Participants

The neuropsychological data were collected in a single-center, cross-sectional rater-blinded study that included patients with definite MS who were undergoing determination of CCSVI status. Exclusion criteria were as follows: (a) presence of relapse or steroid treatment in the 30 days preceding study entry; (b) pre-existing medical conditions known to be associated with brain pathology; (c) pre-existing neuropsychiatric conditions known to be associated with cognitive impairment, including, for example, learning disability, major depressive disorder, schizophrenia and traumatic brain injury, among others; (d) history of cerebral congenital vascular malformations; (e) current alcohol or drug abuse; and (f) pregnancy. Participants underwent a clinical and neuropsychological examination, as well as both trans- and extra-cranial DS. Demographic and clinical information on all participating subjects was acquired using a structured questionnaire and by examination. The collected data included age, sex, age at disease onset, age at diagnosis, symptoms at disease onset and diagnosis, disease duration, Expanded Disability Status Scale (EDSS) [28], disease subtype [29] and the results of physical examination.

The study was approved by the Institutional Review Board and informed consent was obtained from all patients.

### Neuropsychological assessment

The neuropsychological examination was performed by trained personnel who were blinded to the subjects’ clinical and CCSVI characteristics. While patients with current major depressive episode were excluded from the study, remitted or minor depression was permitted, and the degree was quantified using the Beck Depression Inventory Fast Screen (BDIFS) [30] which has been validated in MS [31].

Next, the Minimal Assessment of Cognitive Function in MS (MACFIMS) battery was administered [32], assessing the full spectrum of cognitive domains known to be affected by MS. The MACFIMS has been tested using large prospective MS samples [4,33] and its psychometric properties have been established through the development of the individual tests and further research on the overall battery [34]. Also, the tests on the MACFIMS correlate well with brain magnetic resonance imaging (MRI) metrics in MS samples [35,36]. The specific tests included are as follows: the oral response - version of the Symbol Digit Modalities Test (SDMT) [37], the Paced Auditory Serial Addition Test (PASAT) [38], the California Verbal Learning Test, 2nd edition (CVLT2) [39], the Brief Visual Memory Test, Revised (BVMTR) [40], the Controlled Oral Word Association Test (COWAT) [41], the Judgment of Line Orientation Test (JLO) [42] and the Delis-Kaplan Executive Function System (DKEFS) Sorting Test [43]. The tests were normalized on the basis of recently published normative data that account for demographics, such as age and education [33].

### Doppler sonography

Extra-and trans-cranial DS was performed on a color-coded DS scanner (MyLab 25; Esaote-Biosound, Irvine, CA, USA) equipped with a 5.0- to 10-Mhz transducer to examine venous return in the internal jugular veins (IJVs) and venous veins (VVs). The DS examination was performed by two trained technologists who were blinded to the subjects’ demographic, clinical and neuropsychological characteristics. The detailed scanning protocol and validation were previously reported [23]. Briefly, the following five VH parameters indicative of CCSVI were investigated: 1) reflux/bidirectional flow in the IJV and/or in the VV in sitting and in supine positions, defined as flow directed towards the brain for a duration of >0.88 second; 2) reflux/bidirectional flow in the deep cerebral veins defined as reverse flow for a duration of 0.5 second in one of the intra-cranial veins; 3) B-mode abnormalities or stenoses in IJVs, defined as a cross-sectional area (CSA) of this vein ≤0.3 cm^2^; 4) flow that is not Doppler-detectable in IJVs and/or VVs despite multiple deep breaths; and 5) reverted postural control of the main cerebral venous outflow pathway by measuring the difference of the CSA of the IJVs in the supine and upright positions. A subject was considered CCSVI-positive if ≥2 VH criteria were fulfilled, as previously proposed [10].

We calculated the VH insufficiency severity score (VHISS) [14,44], defined as a weighted sum of the scores contributed by each individual VH criterion. The formula for the VHISS calculations is: VHISS = VHISS1 + VHISS2 + VHISS3 + VHISS4 + VHISS5. The VHISS score is an ordinal measure of the overall extent and number of VH flow pattern anomalies, with a higher value of VHISS indicating a greater severity of abnormal flow. The minimum possible VHISS value is 0 and the maximum 16.

### Statistical analyses

Statistical analyses were performed using SPSS software. As noted above, for descriptive purposes, the raw test scores derived from neuropsychological examination were normalized using previously published data [33]. Partial correlations were performed using the Pearson product–moment correlation coefficient, and the CCSVI positive and negative groups were compared using analysis of variance (or covariance) and chi-square tests. Linear regression was used to examine the hypothesis that CCSVI status as measured by the VHISS score is related to cognitive function or depression, after controlling for education and gender. Throughout, we employed a conservative threshold of P <0.01 to control for type 1 error.

## Results

Of the 109 patients enrolled, 79 were diagnosed with relapsing-remitting, 23 with secondary-progressive, and 7 with primary-progressive disease subtype. All were Caucasian, except two African-Americans and two of Latin American heritage. The other descriptive statistics including demographic, clinical, depression and cognitive outcomes are presented in Table 1. The CCSVI positive and negative MS groups were well matched, and no age, disease duration, EDSS or disease subtype differences were found. While the pattern of cognitive impairment was the same as described in previous studies (SDMT and BVMTR most sensitive), overall we found less impairment in this sample as compared to some previous studies using the same test battery [7,45-47].

**Table 1:** Demographic, clinical and neuropsychological characteristics in multiple sclerosis patients with positive and negative diagnosis of chronic cerebrospinal venous insufficiency (CCSVI)

	**All patients**					**CCSVI positive**		**CCSVI negative**	
						**number = 64**		**number = 45**	
	**Median**	**Mean**	**SD**	**Range**	**Mean z**	**Mean**	**SD**	**Mean**	**SD**
Age	48	47.3	9.8	25 - 66	---	46.8	9.0	47.6	10.4
Education	16	16.0	2.3	12 - 20	---	15.8	2.3	16.1	2.3
Male/female	38/72				---	27/38		11/34	
Disease duration in years	9	10.5	8.2	01 - 41	---	10.6	8.5	10.5	8.1
Disease course: RR/SP/PP	79/23/07				---	48/13/03		31/10/04	
EDSS	2.5	3.3	1.9	0 - 7.5	---	3.2	1.9	3.5	2.0
Beck Depression Inventory Fast Screen	1	2.4	2.6	0 - 16	---	2.5	2.8	2.4	2.4
Symbol Digit Modalities Test	53	53.5	11.7	9 - 74	−0.62	54.9	12.0	51.5	11.3
Paced Auditory Serial Addition Test	48	44.3	13.8	0 - 60	−0.41	45.5	12.6	42.7	15.6
CVLT2Total Learning	55	54.0	11.1	23 - 87	0.37	55.3	12.4	52.1	8.6
CVLT2Delayed Recall	12	11.5	3.2	2 - 16	0.17	11.7	3.5	11.1	2.7
BVMTR Total Learning	23	22.1	6.7	4 - 34	−0.32	22.9	6.8	20.9	6.6
BVMTR Delayed Recall	9	8.6	2.4	1 - 12	−0.63	9.0	2.3	7.9	2.5
Controlled Oral Word Association Test	37	39.1	10.9	14 - 64	0.26	39.6	11.1	38.0	10.7
Judgment of Line Orientation Test	26	24.9	3.9	12 - 30	−0.59	25.6	3.4	24.0	4.6
DKEFS Sorting Correct Sorts	11	10.7	2.2	3 - 15	−0.07	10.7	2.3	10.7	2.0
DKEFS Sorting Description Score	40	40.3	8.7	10 - 58	-0.04	40.4	9.2	40.1	7.9

*BVMTR*, Brief Visuospatial Memory Test, Revised, *CVLT2*, California Verbal Learning Test, 2nd edition, *DKEFS*, Delis-Kaplan Executive Function System, *EDSS*, Expanded disability status scale, *PP*, Primary progressive, *RR*, Relapsing-remitting, *SP*, Secondary progressive, *SD* standard deviation.

**Figure 1:**
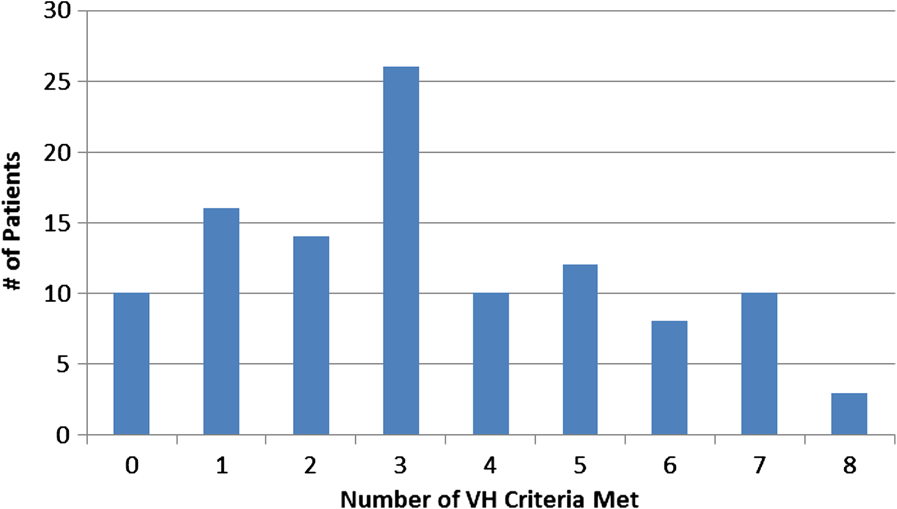
Frequency distribution of venous hemodynamic insufficiency severity score (VHISS) in 109 multiple sclerosis (MS) patients.

There were 64 (58.7%) patients considered CCSVI-positive and 45 negative (Table 1). As shown in Figure 1, the total criteria VHISS score ranged 0 to 8. The median was represented by 26 patients achieving a score of 3.

There were modest trends toward linear correlation between education (r = 0.25) and gender (r = −0.15) and the VHISS score. The chi-square test showed correspondence between gender and the CCSVI-positive diagnosis (*P* = 0.05). Therefore, education and gender were controlled for in hypothesis testing models. There was no correspondence between CCSVI and depression as measured by the BDIFS.

The regression models predicting VHISS score after controlling for education and gender were not statistically significant for any of the MACFIMS predictor variables. The largest partial r in the analysis was −0.13 between CVLTR Delayed Recall and the VHISS score (Table 2).

**Table 2:** Correlation coefficients between venous hemodynamic insufficiency severity score (VHISS) and depression and cognition scores in multiple sclerosis patients (n = 109)

	**Zero order r**	**Partial r**
Beck Depression Inventory Fast Screen	0.10	0.13
Symbol Digit Modalities Test	−0.03	−0.02
Paced Auditory Serial Addition Test	−0.04	−0.07
CVLT2Total Learning	−0.06	−0.04
CVLT2Delayed Recall	−0.13	−0.13
BVMTR Total Learning	0.06	0.04
BVMTR Delayed Recall	0.10	0.09
Controlled Oral Word Association Test	0.02	−0.03
Judgment of Line Orientation Test	0.13	0.00
DKEFS Sorting Correct Sorts	0.04	−0.05
DKEFS Sorting Description Score	0.08	−0.01

*BVMTR* Brief Visuospatial Memory Test, Revised, *CVLT2* California Verbal Learning Test, 2nd edition, *DKEFS* Delis-Kaplan Executive Function System.

**Figure 2:**
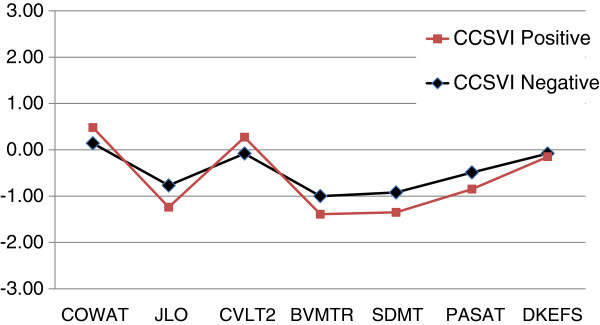
**Cognitive profiles of chronic cerebrospinal venous insufficiency (CCSVI) negative (n = 45) and positive (n = 64) multiple sclerosis patients.** Each value represents a z score based on previously published normative data accounting for demographic variables. There are no significant differences for any test.

The analysis of covariance (ANCOVA) tests showed a significant effect of CCSVI-positive diagnosis on cognitive ability in one of the 10 MACFIMS outcomes (Table 1, Figure 2). For BVMTR Delayed Recall, CCSVI-positive patients showed better performance than their CCSVI-negative counterparts (*P* = 0.009). The direction of the effect was thus counter to expectation in that positive patients achieved a score of 9.1 compared to 7.8 for the CCSVI negative group.

## Discussion

To the best of our knowledge, this is the first investigation of neuropsychological status in MS patients in relation to CCSVI. In this sample of 109 MS patients, we find no evidence of an association between CCSVI and cognitive impairment. Moreover, no relationship between cognitive performance and the severity of CCSVI criteria, as determined by DS, was detected in linear regression analysis. When patients were categorized by their CCSVI status (positive/negative), significant group differences emerged for only one test, in a direction contrary to the hypothesis that CCSVI is a risk factor for cognitive impairment in MS. Similarly, there was no relationship between CCSVI and depression in this cohort.

The CCSVI hypothesis has provoked great controversy and debate in the MS research community since it was first presented [11,13]. The hypothesis gained popularity among MS patients because of the postulated possibility of venous insufficiency correction using endovascular procedures. While the diagnosis of CCSVI can be established using noninvasive and invasive imaging techniques [12], the validity of DS to establish the diagnosis of CCSVI remains controversial. We showed previously that DS, in properly trained hands, has high sensitivity and specificity for CCSVI diagnosis, when compared to invasive imaging methods [48,49]. This was the same method as used in this study, and thus we are confident in the validity of the CCSVI categorization in our analysis.

The true prevalence of CCSVI in MS patients is unknown, and there is good evidence that the condition is also found in patients with other neurologic diseases [23]. In this study, 64% of the participating MS subjects presented with CCSVI, which is similar to our previous study [23]. The difference between the prevalence rates of CCSVI-positive versus -negative MS patients in this study is modest, and of uncertain meaning with respect to MS pathology. Indeed, emerging studies point against CCSVI having a primary causative role in the development of MS [11,13]. A multimodal approach will likely be needed to determine the extent to which CCSVI is present in various healthy and disease groups and MS subtypes [15].

Cognitive impairment is common in MS and can be reliably quantified using neuropsychological tests emphasizing episodic memory, mental processing speed and some aspects of executive function [50]. Neuropsychological deficits are also robustly correlated with brain MRI measures, especially global and regional brain atrophy [51]. The heterogeneity of neuropsychological presentation among MS patients is influenced by many factors, including genetics, gender, intelligence, disease course, comorbid neuropsychiatric illness and health behaviors. The present study employed consensus standard tests emphasizing multiple domains of cognitive function, allowing us to test, in a comprehensive way, whether the presence and severity of CCSVI can influence this important sphere of disability in MS patients. No association between cognitive impairment and the presence and severity of CCSVI was found. This is consistent with our previous findings of a lack of association between the presence of CCSVI and severity of lesion burden and brain atrophy outcomes in MS patients [27].

There are a number of potential limitations in this study. Selection of participants was based on the inclusion or exclusion criteria in patients agreeing to undergo cognitive testing. However, it may be that the most severe patients presenting in our Center were not included in the study. Another potential limit is not including a control group. However, the aim of this study was not to assess CCSVI prevalence, but rather an association with cognitive impairment. Finally, the diagnosis of CCSVI was not confirmed by the use of other invasive diagnostic methods.

## Conclusions

In conclusion, we find no evidence of an association between the presence and severity of CCSVI and cognitive impairment and depression in patients with MS.

## Abbreviations

CCSVI: Chronic cerebrospinal venous insufficiency; DS: Doppler sonography; IJV: Internal jugular vein.

## Competing interests

RHBB receives royalties from Psychological Assessment Resources that are in part associated with the Brief Visuospatial Memory Test Revised. RHBB has acted as a consultant or scientific advisory board member for Bayer, Biogen Idec, Actelion, and Novartis. He has received financial support for research activities from Shire Pharmaceuticals, Accorda and Biogen Idec. BW-G has participated in speaker’s bureaus and served as a consultant for Biogen Idec, Teva Neurosciences, EMD Serono, Pfizer, Novartis, Genzyme, and Acorda. She also has received grant/research support from the agencies listed above as well as ITN, Questcor and Shire. No other industry financial relationships exist. DH has received speaker honoraria and consultant fees from Biogen Idec, Teva Pharmaceutical Industries Ltd., EMD Serono, Pfizer Inc, and Genzyme. RZ has received financial support for research activities from Biogen Idec, Teva Pharmaceutical and Teva Neuroscience, EMD Serono, Genzyme-Sanofi, Novartis, Greatbatch, Bracco and Questcor. He also received personal compensation from Teva Pharmaceutical, Biogen Idec, Novartis, Genzyme-Sanofi, EMD Serono, Bayer, Novartis and General Electric for speaking and consultant services. KM, VV, CK, EC and CB declare that they have no competing interests.

## Authors’ contributions

All authors participated in discussion and correspondence to develop this consensus opinion on the topics covered in this article. RHBB and RZ are the lead authors because they lead the project and wrote the first draft of the manuscript. All authors read and approved the final manuscript.

## Pre-publication history

The pre-publication history for this paper can be accessed here:

http://www.biomedcentral.com/1741-7015/11/167/prepub
